# Mild Cognitive Impairment Staging Yields Genetic Susceptibility, Biomarker, and Neuroimaging Differences

**DOI:** 10.3389/fnagi.2020.00139

**Published:** 2020-06-05

**Authors:** Elizabeth E. Moore, Dandan Liu, Kimberly R. Pechman, Lealani Mae Y. Acosta, Susan P. Bell, L. Taylor Davis, Kaj Blennow, Henrik Zetterberg, Bennett A. Landman, Matthew S. Schrag, Timothy J. Hohman, Katherine A. Gifford, Angela L. Jefferson

**Affiliations:** ^1^Vanderbilt Memory & Alzheimer’s Center, Department of Neurology, Vanderbilt University Medical Center, Nashville, TN, United States; ^2^Department of Biostatistics, Vanderbilt University Medical Center, Nashville, TN, United States; ^3^Division of Cardiovascular Medicine, Department of Medicine, Vanderbilt University Medical Center, Nashville, TN, United States; ^4^Department of Radiology and Radiological Sciences, Vanderbilt University Medical Center, Nashville, TN, United States; ^5^Department of Psychiatry and Neurochemistry, Institute of Neuroscience and Physiology, The Sahlgrenska Academy at University of Gothenburg, Mölndal, Sweden; ^6^Clinical Neurochemistry Laboratory, Sahlgrenska University Hospital, Mölndal, Sweden; ^7^Department of Neurodegenerative Disease, UCL Institute of Neurology, London, United Kingdom; ^8^UK Dementia Research Institute at UCL, London, United Kingdom; ^9^Department of Biomedical Engineering, Vanderbilt University, Nashville, TN, United States; ^10^Department of Electrical Engineering and Computer Science, Vanderbilt University, Nashville, TN, United States; ^11^Vanderbilt Genetics Institute, Vanderbilt University Medical Center, Nashville, TN, United States

**Keywords:** mild cognitive impairment, clinical staging, cerebrospinal fluid, apolipoprotein E, brain MRI

## Abstract

**Introduction:**

While Alzheimer’s disease (AD) is divided into severity stages, mild cognitive impairment (MCI) remains a solitary construct despite clinical and prognostic heterogeneity. This study aimed to characterize differences in genetic, cerebrospinal fluid (CSF), neuroimaging, and neuropsychological markers across clinician-derived MCI stages.

**Methods:**

Vanderbilt Memory & Aging Project participants with MCI were categorized into 3 severity subtypes at screening based on neuropsychological assessment, functional assessment, and Clinical Dementia Rating interview, including mild (*n* = 18, 75 ± 8 years), moderate (*n* = 89 72 ± 7 years), and severe subtypes (*n* = 18, 78 ± 8 years). At enrollment, participants underwent neuropsychological testing, 3T brain magnetic resonance imaging (MRI), and optional fasting lumbar puncture to obtain CSF. Neuropsychological testing and MRI were repeated at 18-months, 3-years, and 5-years with a mean follow-up time of 3.3 years. Ordinary least square regressions examined cross-sectional associations between MCI severity and apolipoprotein E (*APOE*)-ε4 status, CSF biomarkers of amyloid beta (Aβ), phosphorylated tau, total tau, and synaptic dysfunction (neurogranin), baseline neuroimaging biomarkers, and baseline neuropsychological performance. Longitudinal associations between baseline MCI severity and neuroimaging and neuropsychological trajectory were assessed using linear mixed effects models with random intercepts and slopes and a follow-up time interaction. Analyses adjusted for baseline age, sex, race/ethnicity, education, and intracranial volume for MRI models.

**Results:**

Stages differed at baseline on *APOE*-ε4 status (early < middle = late; *p*-values < 0.03) and CSF Aβ (early > middle = late), phosphorylated and total tau (early = middle < late; *p*-values < 0.05), and neurogranin concentrations (early = middle < late; *p*-values < 0.05). MCI stage related to greater longitudinal cognitive decline, hippocampal atrophy, and inferior lateral ventricle dilation (early < late; *p*-values < 0.03).

**Discussion:**

Clinician staging of MCI severity yielded longitudinal cognitive trajectory and structural neuroimaging differences in regions susceptible to AD neuropathology and neurodegeneration. As expected, participants with more severe MCI symptoms at study entry had greater cognitive decline and gray matter atrophy over time. Differences are likely attributable to baseline differences in amyloidosis, tau, and synaptic dysfunction. MCI staging may provide insight into underlying pathology, prognosis, and therapeutic targets.

## Background

Clinicians often categorize Alzheimer’s disease (AD) into severity stages to account for heterogeneity in clinical presentation and progression. However, mild cognitive impairment (MCI), the prodromal phase of AD, remains a singular construct despite many individuals reverting to normal or remaining stable (Ganguli et al., 2004) while others convert to dementia (Ritchie et al., 2001). Prior work has investigated the heterogeneity underlying different stages of MCI (Wu et al., 2012; Lee et al., 2014; Ye et al., 2014; Edmonds et al., 2019), primarily defined as early or late stage based on the Clinical Dementia Rating (CDR) (Morris, 1993), Mini-Mental State Exam (Folstein et al., 1975), and Weschler Memory Scale-Revised Logical Memory Delayed Recall scores (Aisen et al., 2010). Participants with late MCI have greater cerebral amyloid-β (Aβ_42_) deposition (Wu et al., 2012; Edmonds et al., 2019), greater cortical thinning (Ye et al., 2014), and reduced brain connectivity (Lee et al., 2014) compared to early MCI. Additionally, cerebrospinal fluid (CSF) and neuroimaging biomarker profiles change across impairment stages. For example, positron emission tomography (PET) data suggest Aβ_42_ accumulation plateaus early in AD development (Jack et al., 2013), whereas tau accumulation continues to rise as clinical symptoms worsen (Mattsson et al., 2017). Thus, there is a need to better understand the biomarker, imaging, and clinical characteristics of each MCI stage to determine how clinical staging may provide insight into different underlying disease processes, pathological changes, and prognosis. Further, recent evidence suggests other pathologies, such as synaptic dysfunction (measured by CSF neurogranin, Thorsell et al., 2010) or axonal injury [measured by CSF neurofilament light (NFL) (Zetterberg et al., 2016)], contribute to the development and progression of MCI (Kester et al., 2015; Kern et al., 2018), but it is unknown if these pathologies differ across stages of clinical impairment.

This study examined whether clinician-driven MCI staging (early, middle, late) corresponded to differences in apolipoprotein-E (*APOE)* ε4 status, CSF biomarkers, longitudinal neuroimaging outcomes, or longitudinal neuropsychological trajectory. In contrast to prior research examining MCI subtypes, we combined baseline comprehensive neuropsychological performance with participant and informant clinical interview information from the CDR and Functional Activities Questionnaire (FAQ) (Pfeffer et al., 1982) to subtype participants. To examine differences in pathology across the MCI spectrum, we used 3-category staging (early, middle, late), rather than a binary approach, as recent work suggests dichotomous staging of MCI based on memory impairment alone may be insufficient to accurately categorize participants according to neuropathological differences (Edmonds et al., 2019). We hypothesized the stages would exhibit *APOE*-ε4, CSF biomarker, longitudinal neuroimaging, and longitudinal neuropsychological differences across the clinical spectrum of MCI severity. Given prior work suggesting CSF Aβ_42_ levels (Bilgel et al., 2018) and *APOE*-ε4 status (Vemuri et al., 2010) may exert adverse effects prior to neurodegeneration, we also hypothesized early and middle MCI would relate to CSF Aβ_42_ concentrations and *APOE*-ε4 status, while late MCI participants closer to the clinical threshold for dementia would have more phosphorylated tau-related injury, including neurodegeneration. Finally, we hypothesized late MCI participants would show greater neuropsychological decline given their more severe baseline phenotype.

## Materials and Methods

### Study Cohort

The Vanderbilt Memory & Aging Project (Jefferson et al., 2016) is a longitudinal observational study investigating vascular health and brain aging, enriched with older adults with MCI (Albert et al., 2011). Cohort inclusion criteria required participants be at least 60 years of age, speak English, have adequate auditory and visual acuity for testing, and have a reliable study partner. At eligibility, participants underwent a comprehensive assessment including a medical history review, a CDR interview with the participant and informant (Morris, 1993), an FAQ (Pfeffer et al., 1982), and a comprehensive neuropsychological protocol. Participants were excluded for a cognitive diagnosis other than normal cognition (NC), early MCI (Aisen et al., 2010), or MCI (Albert et al., 2011), MRI contraindication, history of neurological disease (e.g., stroke), heart failure, major psychiatric illness, head injury with loss of consciousness > 5 min, or a systemic or terminal illness affecting follow-up participation. The baseline cohort consists of 335 participants, including 132 participants with MCI. Clinicians further categorized MCI participants into three severity subtypes using the CDR, FAQ, and neuropsychological profile. See Table 1 for the clinical staging criteria details. Briefly, early MCI was defined as a CDR global score of 0 or 0.5, having difficulty performing 3 or fewer independent activities of daily living but continuing to complete all activities independently, and neuropsychological impairment in one domain (e.g., memory) or mild impairment in two domains (e.g., memory and executive function). If the latter neuropsychological profile was present (i.e., impairment in more than one domain), the CDR global score had to be 0 and no reported difficulty performing independent activities of daily living (i.e., FAQ = 0). Middle MCI was defined as a CDR global score of 0.5, having difficulty performing 7 or fewer independent activities of daily living but continuing to complete all activities independently, and neuropsychological impairment in memory with possible modest impairment in a second cognitive domain. Late MCI was defined as a CDR global score of 0.5, having difficulty performing at least four independent activities of daily living, requiring at most modest assistance for one or more activities (FAQ score of 2 or 3), and neuropsychological impairment in memory and in a second domain. Neuropsychological impairment in any domain was defined as standard scores falling outside 1.5 standard deviations of age-adjusted normative means (Aisen et al., 2010).

**TABLE 1:** Clinician MCI staging criteria.

	Early MCI	Middle MCI	Late MCI
	*n* = 18	*n* = 89	*n* = 18
CDR	0 or 0.5	0.5	0.5
FAQ	Difficulty in ≤3 activities, but still independent	Difficulty in ≤7 activities, but still independent	Difficulty in ≥4 activities, requires modest assistance
Neuropsychological Impairment	Impairment in 1 or mild impairment in 2*	Impairment in 1 or more domain, must include memory	Impairment in memory and 1 additional domain

*If mild impairment in 2 domains, CDR and FAQ must be 0. Neuropsychological impairment in any domain was defined as standard scores falling outside 1.5 standard deviations of the age-adjusted normative means. CDR, Clinical Dementia Rating; FAQ, Functional Activities Questionnaire; MCI, mild cognitive impairment.

At enrollment, participants completed a comprehensive examination including (but not limited to) fasting blood draw, physical examination, clinical interview, multi-modal brain MRI, neuropsychological assessment, and optional lumbar puncture. Since the purpose of this study was to assess differences across stages of MCI, participants were excluded from the current study for a diagnosis other than MCI. Participants were also excluded for not having complete datasets. See Supplementary Figure 1 for details. Serial neuropsychological assessment and brain MRI were performed at 18-month, 3-year, and 5-year follow up (for which data collection is ongoing). The protocol was approved by the Vanderbilt University Medical Center Institutional Review Board. Written informed consent was obtained prior to data collection.

### Lumbar Puncture and Biochemical Analyses

A subset of participants completed an optional morning fasting lumbar puncture at enrollment (*n* = 55, Supplementary Figure 1). CSF was collected with polypropylene syringes using a Sprotte 25-gauge spinal catheter. Samples were immediately centrifuged, aliquoted into 0.5 mL polypropylene tubes, and stored at −80°C. Samples were analyzed in single batch, and commercially available enzyme-linked immunosorbent assays (Fujirebio, Ghent, Belgium) were used to measure CSF concentrations of Aβ_42_ [INNOTEST^®^ β-AMYLOID_(1–42)_], phosphorylated tau [p-tau, INNOTEST^®^ PHOSPHO-TAU_(181P)_], total tau (t-tau, INNOTEST^®^ hTAU), neurogranin (Kvartsberg et al., 2015), and NFL (Uman Diagnostics). Board certified laboratory technicians processed samples blinded to clinical information (Palmqvist et al., 2014). Intra-assay coefficients of variation were < 10% (Kvartsberg et al., 2015).

### Neuropsychological Protocol

At baseline and each follow-up visit, all participants completed a common neuropsychological protocol assessing language, attention, information processing speed, executive functioning, visuospatial skills, and episodic memory as summarized in Table 2 (Kresge et al., 2018). Measures were carefully selected to preclude floor or ceiling effects and the protocol is distinct from the protocol at eligibility.

**TABLE 2:** Participant demographic, genetic, neuropsychological, and neuroimaging characteristics in the whole sample.

	Total *n* = 125	Early MCI *n* = 18	Middle MCI *n* = 89	Late MCI *n* = 18	*p*-value
**Demographic and Genetic Characteristics**					
Age, years	73 ± 8	75 ± 8	72 ± 7	78 ± 8	**0.005**^†^
Sex, % female	44	56	43	39	0.54
Race, % Non-Hispanic White	85	83	83	94	0.47
Education, years	15 ± 3	15 ± 3	15 ± 3	14 ± 3	0.20
*APOE*-ε4, % carrier	42	11	47	44	**0.02**^‡^
Montreal Cognitive Assessment	23 ± 3	25 ± 3	23 ± 3	20 ± 3	**<0.001**^†‡§^
Follow-up Time, years	3.3 ± 1.8	3.7 ± 1.8	3.3 ± 1.8	3.0 ± 1.7	0.61
Neuropsychological Characteristics*					
Boston Naming Test	26 ± 4	27 ± 2	26 ± 4	24 ± 3	**0.04**^†§^
Animal Naming	16 ± 5	20 ± 5	17 ± 5	12 ± 5	**<0.001**^†‡§^
WAIS-IV Digit-Symbol Coding	46 ± 12	50 ± 10	47 ± 13	38 ± 6	**<0.001**^†§^
DKEFS Number Sequencing, s	51 ± 23	43 ± 15	49 ± 22	64 ± 29	**0.03**^†§^
Executive Function Composite	−0.6 ± 0.9	−0.1 ± 0.8	−0.5 ± 0.9	−1.2 ± 1.0	**0.001**^†‡§^
Hooper Visual Organization Test	23 ± 4	24 ± 3	24 ± 3	20 ± 4	**0.002**^†§^
Memory Composite	−0.7 ± 0.7	−0.1 ± 0.7	−0.8 ± 0.7	−1.4 ± 0.5	**<0.001**^†‡§^
Subjective Cognitive Complaint	354 ± 97	281 ± 85	355 ± 86	417 ± 116	**0.005**^‡§^
**Gray Matter Variables**					
Total gray matter volume, mm^3^	673,559 ± 72,057	645,027 ± 63,578	682,739 ± 73,796	656,248 ± 63,137	0.10
Frontal lobe volume, mm^3^	220,875 ± 28,431	210,911 ± 22,981	224,341 ± 29,791	213,481 ± 23,253	0.11
Temporal lobe volume, mm^3^	132,265 ± 14,748	126,652 ± 12,399	134,258 ± 14,732	127,894 ± 15,411	0.07
Parietal lobe volume, mm^3^	127,361 ± 14,988	121,332 ± 12,559	128,405 ± 15,725	128,285 ± 12,532	0.23
Occipital lobe volume, mm^3^	90,114 ± 10,751	86,602 ± 10,586	91,178 ± 10,695	88,429 ± 10,813	0.29
Hippocampal volume, mm^3^	6891 ± 834	6923 ± 901	6983 ± 834	6409 ± 826	**0.04**^†^
Inferior lateral ventricle volume, mm^3^	2362 ± 1427	1857 ± 874	2319 ± 1464	3078 ± 1473	**0.03**^†§^
AD signature, mm	2.3 ± 0.1	2.3 ± 0.2	2.3 ± 0.2	2.2 ± 0.1	**0.003**^†§^
Intracranial volume, cm^3^	1,505,225 ± 152,389	1,457,056 ± 144,065	1,520,802 ± 157,039	1,477,239 ± 128,520	0.28
**White Matter Variables**					
Total raw WMHs, cm^3^	16.7 ± 19.9	11.4 ± 13.4	16.0 ± 20.1	26.0 ± 22.5	**0.03**^†§^
Total WMHs, log-transformed cm^3^	2.4 ± 1.0	2.0 ± 1.1	2.3 ± 1.0	3.0 ± 0.9	**0.03**^†§^
Frontal lobe WMHs, log-transformed cm^3^	1.8 ± 1.0	1.6 ± 1.0	1.8 ± 1.0	2.4 ± 0.9	**0.05**^†§^
Temporal lobe WMHs, log-transformed cm^3^	0.4 ± 0.5	0.3 ± 0.5	0.4 ± 0.6	0.5 ± 0.4	0.13
Parietal lobe WMHs, log-transformed cm^3^	1.0 ± 1.0	0.8 ± 0.8	1.0 ± 1.0	1.6 ± 1.1	0.06
Occipital lobe WMHs, log-transformed cm^3^	1.2 ± 0.7	0.9 ± 0.7	1.2 ± 0.7	1.4 ± 0.6	0.07
Intracranial volume, cm^3^	1383 ± 148	1337 ± 137	1398 ± 152	1355 ± 133	0.32

*Values denoted as mean ± standard deviation or frequency. Participant characteristics were compared across MCI stages using Kruskal–Wallis test for continuous variables and Pearson test for categorical variables. ^∗^All neuropsychological performance values are total correct excluding times tasks represented by s, seconds. Bold values indicate there is a significant difference between the three stages. ^†^Middle different than late. ^‡^Early different than middle. ^§^ Early different than late. AD, Alzheimer’s disease; APOE, apolipoprotein E; DKEFS, Delis-Kaplan Executive Function System; WAIS, Wechsler Adult Intelligence Scale, 4th Edition; WMHs, white matter hyperintensities.*

### Brain MRI Acquisition and Post-processing

Participants were scanned at the Vanderbilt University Institute of Imaging Science on a 3T Philips Achieva system (Best, Netherlands) using an 8-channel SENSE reception coil array at baseline, 18-month, and 3-year follow-up. A 32-channel coil was used for part of 3-year and all of 5-year data collection.

*T*_1_-weighted images (repetition time = 8.9 ms, echo time = 4.6 ms, spatial resolution = 1 mm × 1 mm ×1 mm) were post-processed with a Multi-Atlas Segmentation pipeline (Asman and Landman, 2013). As previously published (Jefferson et al., 2016), *T*_1_-weighted images were registered to Montreal Neurological Institute (MNI) space, appropriate atlases were registered to *T*_1_-weighted images, and labels were statistically fused. Total intracranial volume and gray matter volume in 7 regions of interests (ROIs), including total, frontal lobe, temporal lobe, parietal lobe, occipital lobe, hippocampal, and inferior lateral ventricle volumes, was calculated.

To evaluate gray matter changes associated with AD pathology (Schwarz et al., 2016), an AD signature variable was calculated. *T*_1_-weighted images were post-processed using FreeSurfer^1^ (Fischl and Dale, 2000). *T*_1_-weighted images were registered to MNI space, intensity corrected, and skull stripped. Subcortical structures, cortical structures, and white matter were segmented, and white and gray matter surfaces were constructed for each hemisphere. These surfaces were used to calculate cortical thickness and inflated for visualization. Surfaces were manually inspected and corrected for registration, topological, and segmentation defects. After manual correction, images were reprocessed to update the transformation template and segmentation information. The AD signature was calculated by summing bilateral cortical thickness measurements from regions shown to distinguish individuals with clinical AD from normal cognition, including the entorhinal cortex, temporal lobe, parietal lobe, fusiform gyrus, and precuneus (Schwarz et al., 2016).

FLAIR images (repetition time = 11000 ms, echo time = 121 ms, spatial resolution = 0.45 mm × 0.45 mm × 4 mm) were post-processed using the Lesion Segmentation Toolbox for Statistical Parametric Mapping (SPM8) (Schmidt et al., 2012). As previously published (Osborn et al., 2018), each *T*_1_-weighted image voxel was defined as gray matter, white matter, or CSF using the SPM8 tissue probability map. FLAIR images were bias-corrected, registered to *T*_1_-weighted images, and manual corrections were made. FLAIR images were segmented into 5 regions of interest (ROIs), including total, frontal, temporal, parietal, and occipital lobe white matter hyperintensity (WMH) volume using an MNI305 lobe atlas (Toro et al., 2009). Intracranial volume was calculated based on a summation of participant-specific gray matter, white matter, and CSF.

### Analytical Plan

*APOE-*ε4 status was defined as carrier (ε2/ε4, ε3/ε4, ε4/ε4) or non-carrier (ε2/ε2, ε2/ε3, ε3/ε3). To assess clinician bias in the classification of MCI severity stages, latent class analysis (LCA) assessed if underlying classes could be statistically determined using the same CDR, FAQ, and neuropsychological testing information at eligibility that defined the clinical MCI stages. The concordance between clinician-derived MCI and LCA-defined stages was tested with Cohen’s kappa.

Prior to cross-sectional analyses, WMHs (cm^3^) were log-transformed due to skewed distribution. In cross-sectional models, logistic regression related clinician-derived MCI severity stages (early, middle, late) to *APOE-*ε4 status. Linear regressions with ordinary least-square estimates related MCI stages to CSF biomarkers, baseline neuroimaging variables, and baseline neuropsychological performance (one test per model). Models adjusted for age, sex, race/ethnicity, education, and intracranial volume for gray matter volume (except the AD signature) and WMH models. In longitudinal models, linear mixed effects regressions with random intercepts and slopes and a follow-up time interaction related baseline MCI severity stage to neuroimaging and neuropsychological trajectory (one test per model). Models adjusted for baseline age, sex, race/ethnicity, education, follow-up time, and baseline intracranial volume for neuroimaging outcomes (except the AD signature). To determine if *APOE*-ε4 frequency differences accounted for results, models were repeated adjusting for *APOE*-ε4 status.

Sensitivity analyses were performed excluding participants with predictor or outcome variables > 4 standard deviations from the group mean, yielding similar results (data not shown). Significance was set *a priori* at *p* < 0.05. The LCA was conducted using Mplus Version 7.3^2^. Remaining analyses were conducted using R 3.5.3^3^.

## Results

### Participant Characteristics

Among all participants (*n* = 125, 73 ± 8 years, range 61–92 years), 18 were classified as early MCI, 89 as middle MCI, and 18 as late MCI by clinical staging criteria. Follow-up time ranged 1.4–5.4 years with a mean of 3.3 years. See Table 2 for characteristics for the entire sample and stratified by MCI severity stage. See Supplementary Table 1 for characteristics for the subset with CSF data.

### Comparison of LCA Outcomes to Clinician-Derived MCI Staging

The LCA was fit to yield three classes, consistent with the clinician-derived MCI stages. Formal concordance testing between the two classification schemes yielded a Cohen’s κ = 0.32, suggesting fair concordance between the clinician-derived staging and statistically derived classifications (Landis and Koch, 1977).

### MCI Stages and *APOE*-ε4 Status

*APOE*-ε4 status differed by MCI stages, such that *APOE*-ε4 carriers were more common among participants with middle (β = 6.04, *p* = 0.02) and late (β = 7.38, *p* = 0.03) compared to early MCI. There was no *APOE*-ε4 status difference between middle and late MCI (β = 1.22, *p* = 0.73).

### MCI Stages and CSF Biomarkers

**FIGURE 1:**
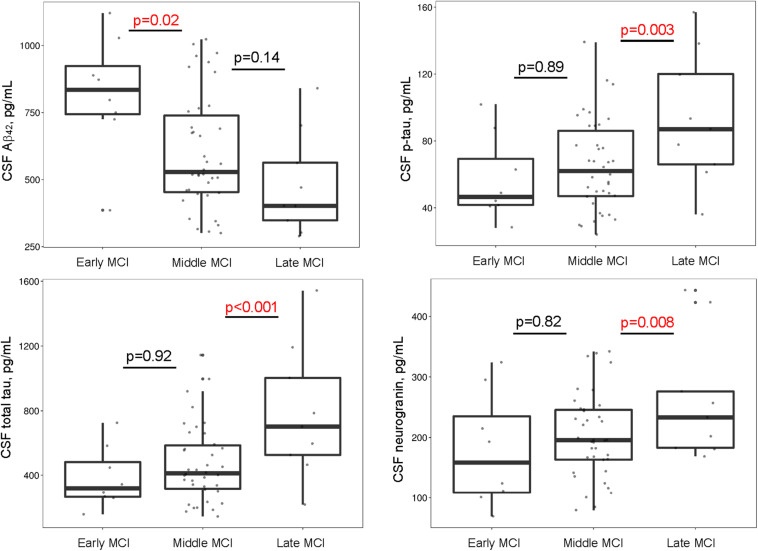
MCI stages differ by CSF biomarkers. Early and middle stages differ on Aβ_42_, such that participants with middle MCI have lower CSF Aβ_42_ concentrations compared to participants with early MCI. Middle and late stages differ on p-tau, total tau, and neurogranin such that participants with late MCI have greater concentrations of CSF p-tau, total tau, and neurogranin compared to participants with middle MCI. Aβ, amyloid-β; CSF, cerebrospinal fluid; MCI, mild cognitive impairment; p-tau, hyperphosphorylated tau.

Between-group comparisons yielded cross-sectional differences in Aβ_42_ (early > middle = late; *p*-values < 0.02), p-tau (early = middle < late; *p*-values < 0.02), t-tau (early = middle < late; *p*-values < 0.005), and neurogranin (early = middle < late; *p*-values < 0.05), such that more advanced stages had greater CSF evidence of pathology. There were no group differences in NFL (*p*-values > 0.11). See Supplementary Table 2 and Figure 1. When adjusting for *APOE*-ε4 status, cross-sectional differences in CSF p-tau, t-tau, and neurogranin concentrations were similar. However, group differences emerged for CSF Aβ_42_ (middle > late; *p* = 0.05) (Supplementary Table 3).

### MCI Stages and Gray Matter

**FIGURE 2:**
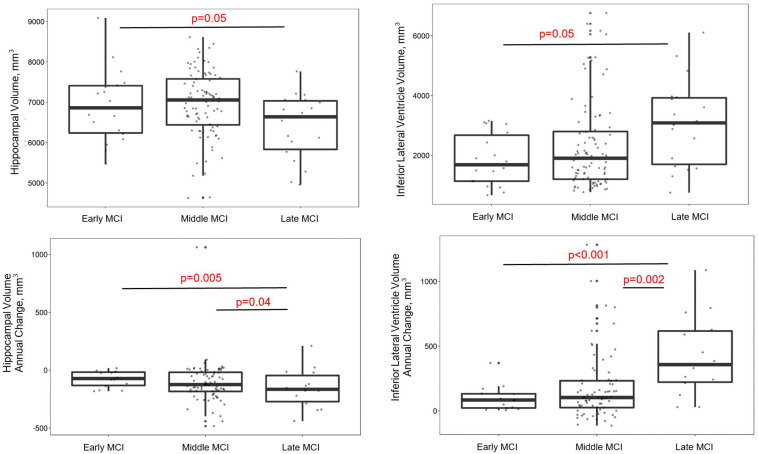
MCI stages differ by gray matter volume in regions susceptible to AD. Early and late stages differ on baseline hippocampal and inferior lateral ventricle volumes, such that late MCI participants have lower hippocampal and higher inferior lateral ventricle volumes than early MCI participants. Late MCI participants also have faster hippocampal atrophy and inferior lateral ventricle dilation compared early and middle MCI participants.

Group differences emerged cross-sectionally for hippocampal volume (early > late; *p* = 0.05), inferior lateral ventricle volume (early < late; *p* = 0.05), and the AD signature (early > late; *p* = 0.03), with greater evidence of atrophy among more advanced stages. However, for parietal lobe volume, participants with late MCI counterintuitively had higher volumes than participants with early (*p* = 0.02) or middle MCI (*p* = 0.03). In longitudinal models, MCI stage related to rate of total gray matter (early < late; *p* = 0.008), parietal lobe (early = middle < late; *p*-values < 0.03), temporal lobe (early < late; *p* = 0.01), and hippocampal atrophy (early = middle < late; *p*-values < 0.04), and inferior lateral ventricle (temporal horn) dilation (early = middle < late; *p*-values < 0.002). See Figure 2 and Table 3 for details. When adjusting for *APOE*-ε4 status, AD signature cross-sectional differences persisted and all longitudinal models were similar (Supplementary Table 4).

**TABLE 3:** MCI stage group differences in cross-sectional and longitudinal neuroimaging outcomes.

	Cross-Sectional		Longitudinal	
	Coefficient	*p*-value	Coefficient	*p*-value
Total WMHs	*F*_2,11__5_ = 2.84	0.06	*F*_2,2__32_ = 0.74	0.48
Early vs. Middle	0.41	0.06	0.006	0.86
Early vs. Late	0.66	**0.02**	0.05	0.28
Middle vs. Late	0.24	0.31	0.05	0.25
Frontal Lobe WMHs	*F*_2,11__5_ = 2.11	0.13	*F*_2,2__32_ = 0.73	0.48
Early vs. Middle	0.30	0.17	0.01	0.67
Early vs. Late	0.59	**0.04**	0.05	0.24
Middle vs. Late	0.28	0.24	0.04	0.29
Temporal Lobe WMHs	*F*_2,11__5_ = 0.68	0.51	*F*_2,2__32_ = 0.99	0.37
Early vs. Middle	0.15	0.26	−0.02	0.43
Early vs. Late	0.15	0.38	0.02	0.61
Middle vs. Late	0.004	0.98	0.04	0.21
Parietal Lobe WMHs	*F*_2,116_ = 2.28	0.11	*F*_2,2__34_ = 0.33	0.72
Early vs. Middle	0.29	0.21	0.01	0.65
Early vs. Late	0.63	**0.03**	0.04	0.42
Middle vs. Late	0.34	0.16	0.02	0.56
Occipital Lobe WMHs	*F*_2,116_ = 2.4	0.10	*F*_2,2__34_ = 0.63	0.54
Early vs. Middle	0.32	**0.04**	0.004	0.88
Early vs. Late	0.32	0.10	0.04	0.32
Middle vs. Late	−0.001	0.99	0.04	0.29
Total Gray Matter Volume	*F*_2,11__5_ = 0.64	0.53	*F*_2,2__32_ = 3.64	**0.03**
Early vs. Middle	11,497	0.26	−3011	0.11
Early vs. Late	8488	0.53	−7274	**0.008**
Middle vs. Late	−3008	0.79	−4263	0.06
Frontal Lobe Volume	*F*_2,11__5_ = 0.52	0.59	*F*_2,2__32_ = 1.37	0.26
Early vs. Middle	5152	0.33	−1210	0.24
Early vs. Late	2097	0.76	−2341	0.11
Middle vs. Late	−3055	0.60	−1131	0.34
Temporal Lobe Volume	*F*_2,11__5_ = 1.20	0.31	*F*_2,2__32_ = 3.39	**0.04**
Early vs. Middle	2707	0.24	−685	0.07
Early vs. Late	−393	0.90	−1343	**0.01**
Middle vs. Late	−3101	0.22	−658	0.13
Parietal Lobe Volume	*F*_2,116_ = 3.03	**0.05**	*F*_2,2__34_ = 3.40	**0.04**
Early vs. Middle	1435	0.54	−502	0.29
Early vs. Late	6927	**0.02**	−1717	**0.01**
Middle vs. Late	5492	**0.03**	−1215	**0.03**
Occipital Lobe Volume	*F*_2,116_ = 0.37	0.69	*F*_2,2__34_ = 1.03	0.36
Early vs. Middle	1158	0.48	−118	0.66
Early vs. Late	80	0.97	−502	0.18
Middle vs. Late	−1077	0.53	−384	0.20
Hippocampal Volume	*F*_2,116_ = 1.92	0.15	*F*_2,2__34_ = 3.96	**0.02**
Early vs. Middle	−173	0.37	−45	0.12
Early vs. Late	−484	**0.05**	−111	**0.005**
Middle vs. Late	−311	0.13	−65	**0.04**
Inferior Lateral Ventricle Volume	*F*_2,116_ = 2.09	0.13	*F*_2,2__34_ = 7.09	**0.001**
Early vs. Middle	505	0.11	113	0.10
Early vs. Late	802	**0.05**	330	**<0.001**
Middle vs. Late	297	0.38	217	**0.002**
AD Signature	*F*_2,114_ = 2.59	0.08	*F*_2,19__6_ = 0.29	0.75
Early vs. Middle	−0.03	0.34	0.004	0.63
Early vs. Late	−0.11	**0.03**	0.008	0.45
Middle vs. Late	−0.07	0.07	0.005	0.62

*Unless otherwise specified, coefficients indicate the β for the corresponding pairwise comparison. Models adjusted for age, sex, race/ethnicity, education, and intracranial volume (except the AD signature). All WMHs were log-transformed prior to cross-sectional analysis. Bold values indicate there is a significant difference. AD, Alzheimer’s disease; WMHs, white matter hyperintensities.*

### MCI Stages and White Matter

MCI stages cross-sectionally differed in the expected direction for total (early < late; *p* = 0.02), frontal lobe (early < late; *p* = 0.04), parietal lobe (early < late; *p* = 0.03), and occipital lobe WMH volume (early < middle; *p* = 0.04), such that more advanced stages have greater WMH volume. In longitudinal models, MCI stage was unrelated to progression of total or regional WMHs. See Table 3 for details. When adjusting for *APOE*-ε4 status, cross-sectional differences remained in total and occipital lobe WMHs and all longitudinal models were similar (Supplementary Table 4).

### MCI Stages and Neuropsychological Performance

As expected, between-group comparisons yielded cross-sectional neuropsychological performance differences in all cognitive domains assessed, such that more advanced stages had worse neuropsychological performance. Specifically, differences emerged for the Boston Naming Test (early > late; *p* = 0.03), Animal Naming (early > middle > late; *p*-values < 0.004), Digit-Symbol Coding (early = middle > late; *p*-values < 0.02), Delis Kaplan Executive Function System (DKEFS) Number Sequencing (early < late; *p* = 0.01), Executive Function Composite (early > middle > late; *p*-values < 0.02), Hooper Visual Organization Test (early = middle > late; *p*-values < 0.001), and Episodic Memory Composite (early > middle > late; *p*-values < 0.001). See Supplementary Table 5 for details.

More importantly, between-group comparisons yielded differences in longitudinal trajectory in the expected direction, such that more advanced stages had greater neuropsychological decline for Boston Naming Test (early = middle < late; *p*-values < 0.008), Animal Naming (early < middle; *p*-value = 0.04), Digit-Symbol Coding (early < late; *p*-value = 0.01), DKEFS Number Sequencing (early = middle < late; *p*-values < 0.004), Executive Function Composite (early < late; *p* = 0.01), and Hooper Visual Organization Test (early = middle < late; *p*-values < 0.003). See Supplementary Table 5 for details. When adjusting for *APOE*-ε4 status, results were similar (Supplementary Table 6).

## Discussion

This study found clinician-derived MCI severity stages yielded differences in *APOE*-ε4 status, CSF biomarkers, neuroimaging outcomes, and neuropsychological trajectory. Specifically, participants with more significant MCI symptoms at study entry had an increased prevalence of the *APOE*-ε4 allele along with greater CSF biomarker evidence of AD pathology (Aβ_42_ and p-tau) and neurodegeneration (t-tau and neurogranin). Late MCI participants also had degeneration of white and gray matter specifically in regions vulnerable to AD and neurodegeneration, as well as faster hippocampal atrophy and inferior lateral ventricle dilation, both signs of AD-type neurodegeneration. Finally, participants with more significant MCI symptoms at study entry had a steeper decline in language, information processing speed, and executive function compared to early MCI participants. Together, these results suggest clinical sub-staging of MCI may provide insight into molecular and structural brain changes and offer valuable prognostic information. Furthermore, the clinician stages were comparable to statistically derived stages suggesting minimal clinician bias and consistency in establishing the categories.

**FIGURE 3:**
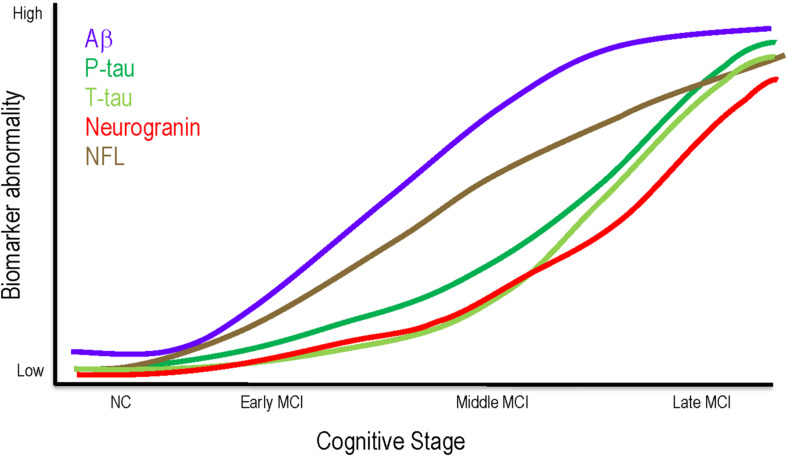
Theoretical model of biomarker changes throughout MCI. Aβ may be most relevant to clinical status in early and middle MCI, plateauing in later stages. Phosphorylated tau, neurodegeneration, and synaptic dysfunction may not exert clinical effects until late MCI. NFL elevations are consistent across all MCI stages. Aβ, amyloid beta; MCI, mild cognitive impairment; NC, normal cognition; NFL, neurofilament light; P-tau, phosphorylated tau; T-tau, total tau.

The current findings contribute to an emerging literature examining the heterogeneity of pathology underlying different stages of MCI (see Figure 3 for a theoretical model incorporating the findings reported here). Middle MCI participants had greater cerebral Aβ_42_ burden and *APOE*-ε4 allele frequency compared to early MCI. However, no differences emerged for these variables when comparing middle and late MCI, indicating these pathologies may be less relevant to clinical status in later stages. Aβ_42_ and *APOE*-ε4 may exert their influence early, prior to the onset of neurodegeneration, consistent with work showing Aβ_42_ deposition plateaus between late MCI and AD development (Jack et al., 2013) and Aβ_42_ status more closely associates with cognitive decline in earlier disease states (Landau et al., 2012). It is possible Aβ_42_ deposition in temporal and frontal regions (Grothe et al., 2017) damages neuronal myelin sheaths (Moore et al., 2018) and disrupts functional connectivity (Yi et al., 2015), leading to cognitive decline prior to the neuroimaging gray matter alterations observed in more severe stages of impairment (Mattsson et al., 2015). Additionally, *APOE*-ε4 may exacerbate early Aβ_42_ deposition (Risacher et al., 2013) via increased aggregation (Liu et al., 2017) or decreased clearance (Castellano et al., 2011), further modifying network connectivity (Wang et al., 2017) and contributing to a phenotype of middle MCI. When accounting for *APOE*-ε4 status, differences between early and middle MCI stages in CSF Aβ_42_ concentrations were attenuated, suggesting the effect of *APOE*-ε4 may be through initial amyloid accumulation.

As clinical impairment worsens, however, other pathologies, such as phosphorylation of tau, synaptic dysfunction, and neurodegeneration may relate more closely associate with clinical stage. Our results show CSF concentrations of p-tau, neurogranin, and t-tau do not differ between early and middle MCI, but late MCI participants have greater concentrations compared to both early and middle MCI participants. Additionally, late MCI participants have greater cross-sectional and longitudinal atrophy in regions susceptible to AD neurodegeneration, including greater hippocampal atrophy, temporal lobe atrophy, and inferior lateral ventricle dilation over time. It is plausible that as Aβ_42_ burden increases in early and middle MCI and possibly worsens tau pathology (Jacobs et al., 2018) and related neurodegeneration, the association between MCI stage and Aβ_42_ burden becomes masked by the robust effects of tau and neurodegeneration on clinical impairment (Brier et al., 2016). As tau deposits in the hippocampus (Braak and Braak, 1991) and induces synaptic dysfunction (Tai et al., 2012), axonal transport deficits (Rodriguez-Martin et al., 2013) and hypometabolism (Wu et al., 2012; Portelius et al., 2015) result in cell death. Thus, greater baseline levels of pathology in late MCI may lead to increased atrophy and steeper cognitive decline over time as observed here. While the results that individuals with late MCI have more tau phosphorylation, synaptic dysfunction, neurodegeneration, and steeper cognitive decline are not surprising, they further validate clinical staging as a valuable metric to detect underlying pathological and prognostic differences.

Contrary to expectation, late MCI participants had greater parietal lobe volume than early and middle MCI participants. It is possible that this increase in volume is due to increased neuroplasticity and activation (Pariente et al., 2005) in regions not yet affected by AD pathology, in response to dysfunctional networks elsewhere (Jacobs et al., 2014). Alternatively, since late MCI participants have lower frontal lobe, temporal lobe, occipital lobe, and hippocampal volumes compared to those with middle MCI, it is possible that if these individuals also had smaller parietal lobes, they would have more severe cognitive consequences warranting a diagnosis of dementia, rather than MCI. Additional studies are needed to further explore this counterintuitive finding.

Notably, MCI stages did not differ on CSF NFL concentrations. While NFL is a marker of axonal injury, CSF NFL concentrations rise due to a variety of pathologies, including demyelination (Malmestrom et al., 2003), amyloid (Mattsson et al., 2019), and tau (Mattsson et al., 2019). Thus, different etiologies may contribute to axonal injury across the MCI spectrum, contributing to the consistent rise in CSF NFL observed here.

Collectively, findings presented here provide evidence that thorough clinical assessment and staging of patients with MCI can provide insight into underlying pathology prior to ordering more expensive and less readily available diagnostic tests and imaging. Specifically, a 3-staging system, validated by unbiased statistical classification, may be more sensitive to changes in underlying Aβ_42_, tau, and concomitant pathologies not detected by dichotomous approaches, allowing clinicians to better predict relevant disease processes underlying cognitive impairment. Clinical staging could lead to more directed diagnostic work-ups and treatment decisions as targeted therapies become available. Given the heterogeneity in MCI conversion to AD (Ritchie et al., 2001; Ganguli et al., 2004), accurate staging will allow clinicians to better provide prognostic information, as cognitive decline and cerebral atrophy occur more rapidly in late compared to early or middle stages of MCI. The ability to predict underlying pathology and progression based on clinical assessment alone could allow for more efficient use of time, money, and clinical resources in the evaluation and of cognitive impairment.

The current study has several strengths, including a clinically well characterized cohort of MCI participants categorized based on comprehensive neuropsychological assessment, CDR, and FAQ. Additionally, the use of a 3-stage paradigm allows for greater investigation into the heterogeneity across the spectrum of MCI, beyond binary group differences between early and late MCI. Comparison to statistically derived stages suggests minimal bias in establishing the clinician staging categories. Finally, the study employed excellent methods for quantifying CSF biomarkers of AD, neurodegeneration, and synaptic dysfunction, gray matter volumes, and WMHs. Core laboratories using quality control procedures analyzed all CSF and MRI measurements in batch, and technicians were blinded to participant clinical details. Despite these strengths, the study is limited by cross-sectional associations with CSF biomarkers and relatively small samples sizes in the early and late MCI stages. Only 44% of participants had CSF data available. While this study has a relatively short follow-up time (3.3 years on average), we would expect longitudinal effects to become more pronounced as follow-up time increases. The majority of the white and gray matter findings would not survive correction for multiple comparisons, raising the possibility of false positive findings. Additionally, the staging protocol, though comprehensive, requires extensive time and an informant is essential for the FAQ and CDR, potentially limiting application in clinical settings. Future work should include longitudinally assessing biomarkers within each MCI stage, examining differences in PET imaging across the MCI spectrum, and determining if an abbreviated version of the staging protocol offers similar value. Importantly, MCI stages differed by age, suggesting results may be partly due to age-related changes. Though models were adjusted for age, replication in larger samples are needed to better delineate age and disease related effects across the MCI spectrum. Finally, the cohort was predominantly non-Hispanic White with participants 61–92 years of age, limiting generalizability to other races, ethnicities, and age groups.

The current study demonstrates novel associations between clinician-derived MCI severity stages and *APOE*-ε4 status, CSF biomarkers of AD pathology and neurodegeneration, cerebral atrophy in regions vulnerable in AD, and cognitive decline. Results support an AD pathogenesis model in which *APOE*-ε4 and Aβ_42_ relate to clinical status in early stages of impairment, whereas phosphorylation of tau, synaptic dysfunction, and neurodegeneration drive more severe impairment, possibly leading to quicker clinical decline. More precise clinical staging of cognitive impairment may provide insights into underlying molecular and structural brain changes, informing diagnostic work-up, treatment, prognosis, and clinical trial recruitment and enrollment strategies.

## Data Availability Statement

The datasets for this manuscript are not publicly available because of participant consent restrictions. Requests to access the datasets should be directed to AJ, angela.jefferson@vumc.org.

## Ethics Statement

The studies involving human participants were reviewed and approved by Vanderbilt University Medical Center Institutional Review Board. The patients/participants provided their written informed consent to participate in this study.

## Author Contributions

EM, KG, and AJ were responsible for conceptualization of the study, acquisition of the data, analysis and interpretation of the data, and drafting the manuscript. MS, KB, HZ, and TH were responsible for analysis and interpretation of the data and drafting the manuscript. KP, LD, and BL were responsible for acquisition of the data and analysis and interpretation of the data. LA and SB were responsible for acquisition of the data. DL was responsible for analysis and interpretation of the data.

## Conflict of Interest

KB has served as a consultant or at advisory boards for Alector, Biogen, CogRx, Lilly, MagQu, Novartis, and Roche Diagnostics, and is a co-founder of Brain Biomarker Solutions in Gothenburg AB, a GU Venture-based platform company at the University of Gothenburg, all unrelated to the work presented in this paper. The remaining authors declare that the research was conducted in the absence of any commercial or financial relationships that could be construed as a potential conflict of interest.

## Footnotes

1 http://surfer.nmr.mgh.harvard.edu/

2 www.statmodel.com

3 www.r-project.org
