# Supplementary material for: Mild Cognitive Impairment Staging Yields Genetic Susceptibility, Biomarker, and Neuroimaging Differences
Source: Front Aging Neurosci. 2020 Jun 5;12:139. doi: 10.3389/fnagi.2020.00139 (PMC7289958; doi:10.3389/fnagi.2020.00139)
Supplement: Supplementary file 1 [file Table_1.DOCX]

**Supplementary Files**

Mild cognitive impairment staging yields genetic susceptibility, biomarker, and neuroimaging differences

**Figure 1:** Participant Inclusion/Exclusion Details

**Table 1.** Participant Demographic and CSF Biomarker and Characteristics in the CSF Sample

**Table 2.** MCI Stage Group Cross-Sectional Differences in CSF Biomarkers

**Table 3.** MCI Stage Group Cross-Sectional Differences in CSF Biomarkers Adjusting for *APOE*-ε4 Status

**Table 4.** MCI Stage Group Differences in Cross-Sectional and Longitudinal Neuroimaging Outcomes Adjusting for *APOE*-ε4 Status

**Table 5.** MCI Stage Group Differences in Cross-Sectional and Longitudinal Neuropsychological Performance

**Table 6.** MCI Stage Group Differences in Cross-Sectional and Longitudinal Neuropsychological Performance Adjusting for *APOE*-ε4 Status



**Figure 1.**

**Figure 1. Participant Inclusion/Exclusion Details.** Missing categories are mutually exclusive. One participant did not have usable neuroimaging data (n=124 in neuroimaging analyses) and an additional 3 participants had AD signature data that was unusable due to quality control parameters (n=121 in AD signature analyses). One participant did not have CSF NFL data (n=54 in NFL analysis). AD=Alzheimer’s disease: CSF=cerebrospinal fluid; MCI=mild cognitive impairment; NFL=neurofilament light.

**Table 1. Participant Demographic and CSF Biomarker and Characteristics in the CSF Sample**

|  | **Total**  **n=55** | **Early MCI**  **n=8** | **Middle MCI**  **n=38** | **Late MCI**  **n=9** | **p-value** |
| --- | --- | --- | --- | --- | --- |
| **Demographic & Genetic Characteristics** |  |  |  |  |  |
| Age, years | 73±6 | 76±7 | 72±6 | 76±6 | 0.14 |
| Sex, % female | 42 | 25 | 50 | 44 | 0.58 |
| Race, % Non-Hispanic White | 91 | 100 | 87 | 44 | 0.29 |
| Education, years | 15±3 | 14±3 | 15±3 | 14±4 | 0.37 |
| *APOE*-ε4, % carrier | 44 | 13 | 45 | 44 | 0.15 |
| Montreal Cognitive Assessment | 23±3 | 26±3 | 24±3 | 19±3 | **<0.001*^†^** |
| **CSF Biomarkers, pg/mL** |  |  |  |  |  |
| Aβ_42_ | 614±229 | 821±222 | 602±214 | 480±188 | **0.01^†‡^** |
| P-tau | 69±31 | 57±26 | 65±27 | 93±39 | 0.07 |
| T-tau | 510±290 | 386±189 | 471±239 | 781±406 | **0.02^†‡^** |
| Neurogranin | 209±82 | 179±94 | 202±69 | 263±103 | 0.17 |
| Neurofilament Light | 1238±720 | 992±328 | 1213±733 | 1562±861 | 0.19 |
| **Note**. Values denoted as mean standard deviation or frequency. Participant characteristics were compared across MCI stages using Kruskal-Wallis test for continuous variables and Pearson test for categorical variables. *Middle different than late. †Early different than late. ‡Early different than middle. Aβ=amyloid beta; AD=Alzheimer’s disease; APOE=apolipoprotein E; P-tau=phosphorylated tau; T-tau=total tau; | | | | | |

**Table 2. MCI Stage Group Cross-Sectional Differences in CSF Biomarkers**

|  | **Coefficient** | **p-value** |
| --- | --- | --- |
| Aβ_42_ | F_2,48_=5.56 | **0.007** |
| Early vs. Middle | -207 | **0.02** |
| Early vs. Late | -328 | **0.002** |
| Middle vs. Late | -120 | 0.14 |
| P-tau | F_2,48_=5.27 | **0.009** |
| Early vs. Middle | -1.6 | 0.89 |
| Early vs. Late | 31.8 | **0.02** |
| Middle vs. Late | 33.4 | **0.003** |
| T-tau | F_2,48_=7.56 | **0.001** |
| Early vs. Middle | -10 | 0.92 |
| Early vs. Late | 353 | **0.005** |
| Middle vs. Late | 363 | **<0.001** |
| Neurogranin | F_2,48_=4.03 | **0.02** |
| Early vs. Middle | -7 | 0.82 |
| Early vs. Late | 70 | **0.048** |
| Middle vs. Late | 76 | **0.008** |
| Neurofilament Light | F_2,47_=1.36 | 0.27 |
| Early vs. Middle | 303 | 0.33 |
| Early vs. Late | 583 | 0.11 |
| Middle vs. Late | 280 | 0.33 |
| **Note**. Analyses performed on n=55 participants. For neurofilament light analyses, n=54. Unless otherwise specified, coefficients indicate the β for the corresponding pairwise comparison. Models adjusted for age, sex, race/ethnicity, and education. Aβ=amyloid beta; p-tau=hyperphosphorylated tau; t-tau=total tau. | | |

**Table 3. MCI Stage Group Cross-Sectional Differences in CSF Biomarkers Adjusting for *APOE*-ε4 Status**

|  | **Coefficient** | **p-value** |
| --- | --- | --- |
| Aβ_42_ | F_2,47_=4.49 | **0.02** |
| Early vs. Middle | -109 | 0.14 |
| Early vs. Late | -241 | **0.005** |
| Middle vs. Late | -133 | **0.05** |
| P-tau | F_2,47_=7.98 | **0.007** |
| Early vs. Middle | -7.6 | 0.51 |
| Early vs. Late | 26.5 | **0.05** |
| Middle vs. Late | 34.1 | **0.002** |
| T-tau | F_2,47_=7.98 | **0.001** |
| Early vs. Middle | -69 | 0.51 |
| Early vs. Late | 302 | **0.01** |
| Middle vs. Late | 371 | **<0.001** |
| Neurogranin | F_2,47_=4.15 | **0.02** |
| Early vs. Middle | -20 | 0.52 |
| Early vs. Late | 58 | 0.10 |
| Middle vs. Late | 78 | **0.006** |
| Neurofilament Light | F_2,46_=1.17 | 0.32 |
| Early vs. Middle | 267 | 0.41 |
| Early vs. Late | 553 | 0.14 |
| Middle vs. Late | 286 | 0.33 |
| **Note**. Analyses performed on n=55 participants. For neurofilament light analyses, n=54. Unless otherwise specified, coefficients indicate the β for the corresponding pairwise comparison. Models adjusted for age, sex, race/ethnicity, education, and *APOE*-ε4 status. APOE=apolipoprotein E; Aβ=amyloid beta; p-tau=hyperphosphorylated tau; t-tau=total tau. | | |

**Table 4. MCI Stage Group Differences in Cross-Sectional and Longitudinal Neuroimaging Outcomes Adjusting for *APOE*-ε4 Status**

|  | **Cross-Sectional** | | **Longitudinal** | |
| --- | --- | --- | --- | --- |
|  | **Coefficient** | **p-value** | **Coefficient** | **p-value** |
| Total WMHs | F_2,114_=2.09 | 0.13 | F_2,232_=0.72 | 0.49 |
| Early vs. Middle | 0.35 | 0.12 | 0.006 | 0.86 |
| Early vs. Late | 0.59 | **0.05** | 0.05 | 0.28 |
| Middle vs. Late | 0.23 | 0.33 | 0.05 | 0.25 |
| Frontal Lobe WMHs | F_2,114_=1.48 | 0.23 | F_2,232_=0.70 | 0.50 |
| Early vs. Middle | 0.23 | 0.30 | 0.01 | 0.67 |
| Early vs. Late | 0.50 | **0.09** | 0.05 | 0.25 |
| Middle vs. Late | 0.27 | 0.26 | 0.04 | 0.30 |
| Temporal Lobe WMHs | F_2,114_=0.48 | 0.62 | F_2,232_=0.99 | 0.37 |
| Early vs. Middle | 0.13 | 0.34 | -0.02 | 0.42 |
| Early vs. Late | 0.13 | 0.46 | 0.02 | 0.62 |
| Middle vs. Late | <0.001 | 1.00 | 0.04 | 0.21 |
| Parietal Lobe WMHs | F_2,115_=1.9 | 0.16 | F_2,234_=0.32 | 0.73 |
| Early vs. Middle | 0.24 | 0.31 | 0.01 | 0.65 |
| Early vs. Late | 0.58 | **0.06** | 0.04 | 0.42 |
| Middle vs. Late | 0.34 | 0.16 | 0.02 | 0.57 |
| Occipital Lobe WMHs | F_2,115_=2.01 | 0.14 | F_2,234_=0.62 | 0.54 |
| Early vs. Middle | 0.30 | **0.05** | 0.004 | 0.88 |
| Early vs. Late | 0.30 | 0.13 | 0.04 | 0.32 |
| Middle vs. Late | -0.001 | 0.99 | 0.04 | 0.29 |
| Total Grey Matter Volume | F_2,114_=0.88 | 0.42 | F_2,232_=3.47 | **0.03** |
| Early vs. Middle | 13,808 | 0.19 | -3142 | 0.10 |
| Early vs. Late | 11,270 | 0.41 | -7072 | **0.009** |
| Middle vs. Late | -2538 | 0.82 | -3930 | 0.08 |
| Frontal Lobe Volume | F_2,114_=0.59 | 0.55 | F_2,232_=1.33 | 0.27 |
| Early vs. Middle | 5693 | 0.30 | -1232 | 0.23 |
| Early vs. Late | 2734 | 0.70 | -2301 | 0.11 |
| Middle vs. Late | -2959 | 0.61 | -1069 | 0.37 |
| Temporal Lobe Volume | F_2,114_=1.5 | 0.23 | F_2,232_=3.41 | 0.03 |
| Early vs. Middle | 3463 | 0.14 | -701 | 0.06 |
| Early vs. Late | 527 | 0.86 | -1329 | **0.01** |
| Middle vs. Late | -2935 | 0.24 | -628 | 0.15 |
| Parietal Lobe Volume | F_2,115_=3.51 | **0.03** | F_2,234_=3.34 | 0.04 |
| Early vs. Middle | 2255 | 0.35 | -510 | 0.28 |
| Early vs. Late | 7774 | **0.01** | -1706 | **0.01** |
| Middle vs. Late | 5520 | **0.03** | -1196 | **0.03** |
| Occipital Lobe Volume | F_2,115_=0.42 | 0.66 | F_2,234_=1.02 | 0.36 |
| Early vs. Middle | 1332 | 0.43 | -120 | 0.66 |
| Early vs. Late | 275 | 0.90 | -502 | 0.18 |
| Middle vs. Late | -1057 | 0.54 | -382 | 0.21 |
| Hippocampal Volume | F_2,115_=1.34 | 0.27 | F_2,234_=3.87 | **0.02** |
| Early vs. Middle | -54 | 0.78 | -45 | 0.12 |
| Early vs. Late | -356 | 0.15 | -110 | **0.006** |
| Middle vs. Late | -302 | 0.13 | -65 | **0.04** |
| Inferior Lateral Ventricle Volume | F_2,115_=1.35 | 0.26 | F_2,234_=7.09 | **0.001** |
| Early vs. Middle | 373 | 0.25 | 113 | 0.10 |
| Early vs. Late | 668 | 0.11 | 330 | **<0.001** |
| Middle vs. Late | 294 | 0.38 | 217 | **0.002** |
| AD Signature | F_2,113_=2.15 | 0.12 | F_2,196_=0.27 | 0.76 |
| Early vs. Middle | -0.02 | 0.51 | 0.004 | 0.64 |
| Early vs. Late | -0.10 | **0.05** | 0.008 | 0.46 |
| Middle vs. Late | -0.07 | 0.07 | 0.004 | 0.64 |
| **Note**. Unless otherwise specified, coefficients indicate the β for the corresponding pairwise comparison. Models adjusted for age, sex, race/ethnicity, education, intracranial volume (except the AD signature), and *APOE*-ε4 status. All WMHs were log-transformed prior to cross-sectional analysis. AD=Alzheimer’s disease; APOE=apolipoprotein E; WMHs=white matter hyperintensities. | | | | |

**Table 5. MCI Stage Group Differences in Cross-Sectional and Longitudinal Neuropsychological Performance**

|  | **Cross-Sectional** | | **Longitudinal** | |
| --- | --- | --- | --- | --- |
|  | **Coefficient** | **p-value** | **Coefficient** | **p-value** |
| Boston Naming Test | F_2,118_=2.69 | 0.07 | F_2,252_=4.50 | **0.01** |
| Early vs. Middle | -1.7 | **0.05** | -0.31 | 0.38 |
| Early vs. Late | -2.4 | **0.03** | -1.34 | **0.006** |
| Middle vs. Late | -0.7 | 0.42 | -1.02 | **0.008** |
| Animal Naming | F_2,118_=14.5 | **<0.001** | F_2,252_=2.42 | 0.09 |
| Early vs. Middle | -4.2 | **<0.001** | -0.71 | **0.04** |
| Early vs. Late | -7.6 | **.001** | -0.83 | 0.07 |
| Middle vs. Late | -3.4 | **0.004** | -0.12 | 0.75 |
| WAIS-IV Digit-Symbol Coding | F_2,118_=3.85 | **0.02** | F_2,248_=3.13 | **0.05** |
| Early vs. Middle | -3.1 | 0.28 | -1.27 | 0.12 |
| Early vs. Late | -10.1 | **0.008** | -2.77 | **0.01** |
| Middle vs. Late | -7.0 | **0.02** | -1.49 | 0.10 |
| DKEFS Number Sequencing, s | F_2,118_=3.23 | **0.04** | F_2,244_=5.57 | **0.004** |
| Early vs. Middle | 7.2 | 0.21 | 0.60 | 0.79 |
| Early vs. Late | 18.7 | **0.01** | 9.47 | **0.004** |
| Middle vs. Late | 11.5 | 0.06 | 8.86 | **0.001** |
| Executive Function Composite | F_2,118_=7.93 | **<0.001** | F_2,236_=3.12 | **0.05** |
| Early vs. Middle | -0.6 | **0.01** | -0.09 | 0.16 |
| Early vs. Late | -1.1 | **<0.001** | -0.22 | **0.01** |
| Middle vs. Late | -0.6 | **0.02** | -0.12 | 0.08 |
| Hooper Visual Organization Test | F_2,118_=7.14 | 0.001 | F_2,252_=5.62 | **0.004** |
| Early vs. Middle | -1.0 | 0.22 | -0.21 | 0.54 |
| Early vs. Late | -3.9 | **<0.001** | -1.35 | **0.003** |
| Middle vs. Late | -2.9 | **0.001** | -1.14 | **0.002** |
| Memory Composite | F_2,118_=19.01 | **<0.001** | F_2,235_=1.27 | 0.28 |
| Early vs. Middle | -0.7 | **<0.001** | -0.03 | 0.42 |
| Early vs. Late | -1.3 | **<0.001** | -0.09 | 0.12 |
| Middle vs. Late | -0.6 | **<0.001** | -0.05 | 0.23 |
| **Note**. Analyses performed on n=125 participants. Unless otherwise specified, coefficients indicate the β for the corresponding pairwise comparison. Models adjusted for age, sex, race/ethnicity, and education. DKEFS=Delis-Kaplan Executive Function System; WAIS=Wechsler Adult Intelligence Scale 4^th^ Edition. | | | | |

**Table 6. MCI Stage Group Differences in Cross-Sectional and Longitudinal Neuropsychological Performance Adjusting for *APOE*-ε4 Status**

|  | **Cross-Sectional** | | **Longitudinal** | |
| --- | --- | --- | --- | --- |
|  | **Coefficient** | **p-value** | **Coefficient** | **p-value** |
| Boston Naming Test | F_2,117_=2.22 | 0.11 | F_2,252_=4.49 | **0.01** |
| Early vs. Middle | -1.5 | 0.08 | -0.31 | 0.38 |
| Early vs. Late | -2.2 | **0.05** | -1.33 | **0.006** |
| Middle vs. Late | -0.7 | 0.43 | -1.02 | **0.008** |
| Animal Naming | F_2,117_=13.12 | **<0.001** | F_2,252_=2.39 | 0.09 |
| Early vs. Middle | -4.0 | **<0.001** | -0.70 | **0.04** |
| Early vs. Late | -7.4 | **<0.001** | -0.82 | 0.08 |
| Middle vs. Late | -3.4 | **0.004** | -0.12 | 0.75 |
| WAIS-IV Digit-Symbol Coding | F_2,117_=3.31 | **0.04** | F_2,248_=3.06 | **0.05** |
| Early vs. Middle | -2.3 | 0.45 | -1.25 | 0.12 |
| Early vs. Late | -9.2 | **0.02** | -2.72 | **0.01** |
| Middle vs. Late | -6.9 | **0.03** | -1.47 | 0.11 |
| DKEFS Number Sequencing, s | F_2,117_=2.90 | 0.06 | F_2,244_=5.57 | **0.004** |
| Early vs. Middle | 6.5 | 0.27 | 0.59 | 0.79 |
| Early vs. Late | 17.9 | **0.02** | 9.41 | **0.004** |
| Middle vs. Late | 11.4 | 0.06 | 8.82 | **0.001** |
| Executive Function Composite | F_2,117_=6.97 | **0.001** | F_2,236_=3.06 | **0.05** |
| Early vs. Middle | -0.52 | **0.02** | -0.09 | 0.16 |
| Early vs. Late | -1.1 | **<0.001** | -0.22 | **0.01** |
| Middle vs. Late | -0.55 | **0.02** | -0.12 | 0.08 |
| Hooper Visual Organization Test | F_2,117_=6.63 | 0.002 | F_2,252_=5.65 | **0.004** |
| Early vs. Middle | -0.87 | 0.31 | -0.21 | 0.54 |
| Early vs. Late | -3.7 | **0.001** | -1.35 | **0.003** |
| Middle vs. Late | -2.8 | **0.002** | -1.14 | **0.002** |
| Memory Composite | F_2,117_=16.92 | **<0.001** | F_2,235_=1.27 | 0.28 |
| Early vs. Middle | -0.67 | **<0.001** | -0.03 | 0.42 |
| Early vs. Late | -1.3 | **<0.001** | -0.09 | 0.11 |
| Middle vs. Late | -0.6 | **<0.001** | -0.05 | 0.23 |
| **Note**. Analyses performed on n=125 participants. Unless otherwise specified, coefficients indicate the β for the corresponding pairwise comparison. Models adjusted for age, sex, race/ethnicity, education, and *APOE*-ε4 status. APOE=apolipoprotein E; DKEFS=Delis-Kaplan Executive Function System; WAIS=Wechsler Adult Intelligence Scale 4^th^ Edition. | | | | |
